# Extreme Phenotypes With Identical Mutations: Two Patients With Same Non-sense *NHEJ1* Homozygous Mutation

**DOI:** 10.3389/fimmu.2018.02959

**Published:** 2019-01-07

**Authors:** Maria J. Recio, Nerea Dominguez-Pinilla, Melina Soledad Perrig, Carmen Rodriguez Vigil-Iturrate, Nerea Salmón-Rodriguez, Cristina Martinez Faci, María J. Castro-Panete, Javier Blas-Espada, Marta López-Nevado, Raquel Ruiz-Garcia, Rebeca Chaparro-García, Luis M. Allende, Luis Ignacio Gonzalez-Granado

**Affiliations:** ^1^Department of Immunology, Ophthalmology and ENT, School of Medicine, Complutense University, 12 de Octubre Health Research Institute (imas12), Madrid, Spain; ^2^Hospital 12 de Octubre Health Research Institute (imas12), Madrid, Spain; ^3^Pediatric Hematology and Oncology Unit, University Hospital Virgen de la Salud, Toledo, Spain; ^4^Pediatric Hematology and Oncology Unit, University Hospital Miguel Servet, Zaragoza, Spain; ^5^Immunodeficiencies Unit, Pediatrics, University Hospital 12 octubre, Madrid, Spain; ^6^Complutense University School of Medicine, Madrid, Spain; ^7^Department of Immunology, University Hospital 12 Octubre, Madrid, Spain

**Keywords:** XLF/Cernunnos, NHEJ1 mutation, DNA repair, severe combined immunodeficiency, lymphomagenesis, radiosensitive SCID (RS-SCID)

## Abstract

Cernunnos/XLF deficiency is a rare primary immunodeficiency classified within the DNA repair defects. Patients present with severe growth retardation, microcephaly, lymphopenia and increased cellular sensitivity to ionizing radiation. Here, we describe two unrelated cases with the same non-sense mutation in the *NHEJ1* gene showing significant differences in clinical presentation and immunological profile but a similar DNA repair defect.

## Introduction

### Clinical Presentation and Laboratory Test Results

We report two patients harboring the same homozygous mutation in *NHEJ1* gene. Strikingly, their clinical phenotypes differed markedly. One presented with severe combined immunodeficiency whereas the other only had isolated thrombocytopenia and macrocytosis. Clinical characteristics are summarized in Table 1. Both patients presented severe lymphopenia: immunophenotyping of Patient 1 (P1) showed severe T and B-cell lymphopenia and normal to elevated NK cells (T^−^B^−^NK^+^ phenotype) in contrast to Patient 2 (P2) who presented a milder immunophenotype. T-cell compartment also showed differences between the two patients: P1 had a more senescent T-cell phenotype with increase in CD4^+^ and CD8^+^ effector memory (CCR7^−^CD45RA^−^) and decrease naïve and recent thymic emigrants (RTE) (CD4+CD45RA+CD31+) T cells and a higher proportion of activated T-cells (CD3^+^HLA-DR^+^) (Table 2). Of note, P1 had a severe decrease in CD8+ population mimicking the one reported in ZAP70 or HLA class I deficiencies among patients with combined immunodeficiency/severe combined immunodeficiency.

**Table 1:** Clinical features of the patients with Cernunnos/XLF deficiency.

		**P1**	**P2**
	Origin	Caucasic	Caucasic
	Consanguinity	No	No
Age	Onset	1 m	9 m
	Current	1 year 10 m.	8 year 4 m.
Clinical features	Microcephaly	+	+
	Growth retardation	+	+
	Facial dysmorphism	–	+
Additional clinical features	Neurological manifestations	–	–
	Bone malformation	–	–
	Autoimmunity	–	+
	Cytopenias	–	+ (thrombocytopenia)
Infections	Respiratory tract infections	-	+
	Bacterial and opportunistic infection	–	–
	Urinary tract abnormalities	–	–
	Age at HSCT	4.5 m. and 9 months	7y. 10 m.
Outcome	Status	Alive and well (HSCT)	Alive and well (HSCT)

**Table 2:** Immunologic features of patients with Cernunnos/XLF deficiency.

**Parameter**	**RefValues (children)**	**P1**	**P2**
Lymphocyte (n°/μL)	2500–6000	809	879
**T CELLS**			
CD3+ n°/μL (%)	1400–4300 (52–88)	60 (7)	661 (75)
CD3+TCRab (%)	85–99	5	54
CD3+TCRγδ (%)	2–15	1	16
CD3+HLA–DR+ (%)	0–10	22	7
CD3+TCRαβCD4–CD8- (%)	0–2.5	0.2	0.7
CD4+ n°/μL (%)	1000–2500 (33–55)	53 (7)	304 (35)
CD4+CD45RA+CCR7+(Naïve) (%)	32–82	4.1	45.4
CD4+CD45RA–CCR7+ (CM) (%)	15–30	41.5	28.9
CD4+CD45RA–CCR7– (EM) (%)	8–30	53.9	23.8
CD4+CD45RA+CCR7– (E) (%)	0.4–4	0.4	1.89
CD4+CD45RA+CD31+ (%)	44–60	2	ND
CD8+ n°/μL (%)	400–1400 (17–34)	6 (1)	264 (30)
CD8+CD45RA+CCR7+(Naïve) (%)	30–80	15.3	72.0
CD8+CD45RA−CCR7+ (CM) (%)	3–28	16.2	4.5
CD8+CD45RA–CCR7– (EM) (%)	17–40	59.5	16.7
CD8+CD45RA+CCR7– (TEMRA) (%)	2–15	9	6.8
TRECS (copies/punch)	> 10	< 10	50
**NK CELLS**			
CD56+CD3– n°/μL (%)	100–650 (2–20)	671 (83)	191 (21.7)
**B CELLS**			
CD19+ n°/μL (%)	400–1500 (9–28)	49 (6)	22 (2.5)
CD19+CD27+ (%)	7–19	ND	32
CD19+IgD+CD27– (%Naive)	75–89	ND	63
CD19+IgD+CD27+ (%MZ)	2.6–7.1	ND	14.9
CD19+IgD–CD27+ (%SW)	4.5–20	ND	17.10
CD19+CD38hiIgM+ (%Transitional)	3–10	ND	13
Plasmablasts	0.5–5	ND	4.6
KRECS (copies/punch)	>10	< 10	100
**SERUM IMMUNOGLOBULINS (mg/dl)**			
IgG (mg/dL)	600–1230	446	779
IgA (mg/dL)	30–200	18	<6.67
IgM (mg/dL)	50–200	40	109
**SPECIFIC ANTIBODIES**			
IgG vs. Pneumococcus (mg/dL)	>5.4	ND	2.9
IgG2 vs. Pneumococcus (mg/dL)	>2.4	ND	0.36
IgG vs. Tetanus toxoid (IU/mL)	>0.1	ND	9.10

*ND, not determined*.

B cells were severely reduced in both patients. The B-cell profile in P2 showed a normal proportion of naive, unswitched memory, switched memory, transitional, and plasmoblast B-cells. Serum immunoglobulins were reduced in P1. Due to age limitation, specific antibody response was only tested in P2, showing a specific antibody deficiency: IgG and IgG2 levels against pneumococcus antigen were reduced while IgG levels against tetanus toxoid antigen showed normal values after vaccination. Regarding to the immunophenotype in T and B-cells, P1 had very low TRECs and KRECs copies, whereas P2 had preserved levels (Table 2).

**Figure 1:**
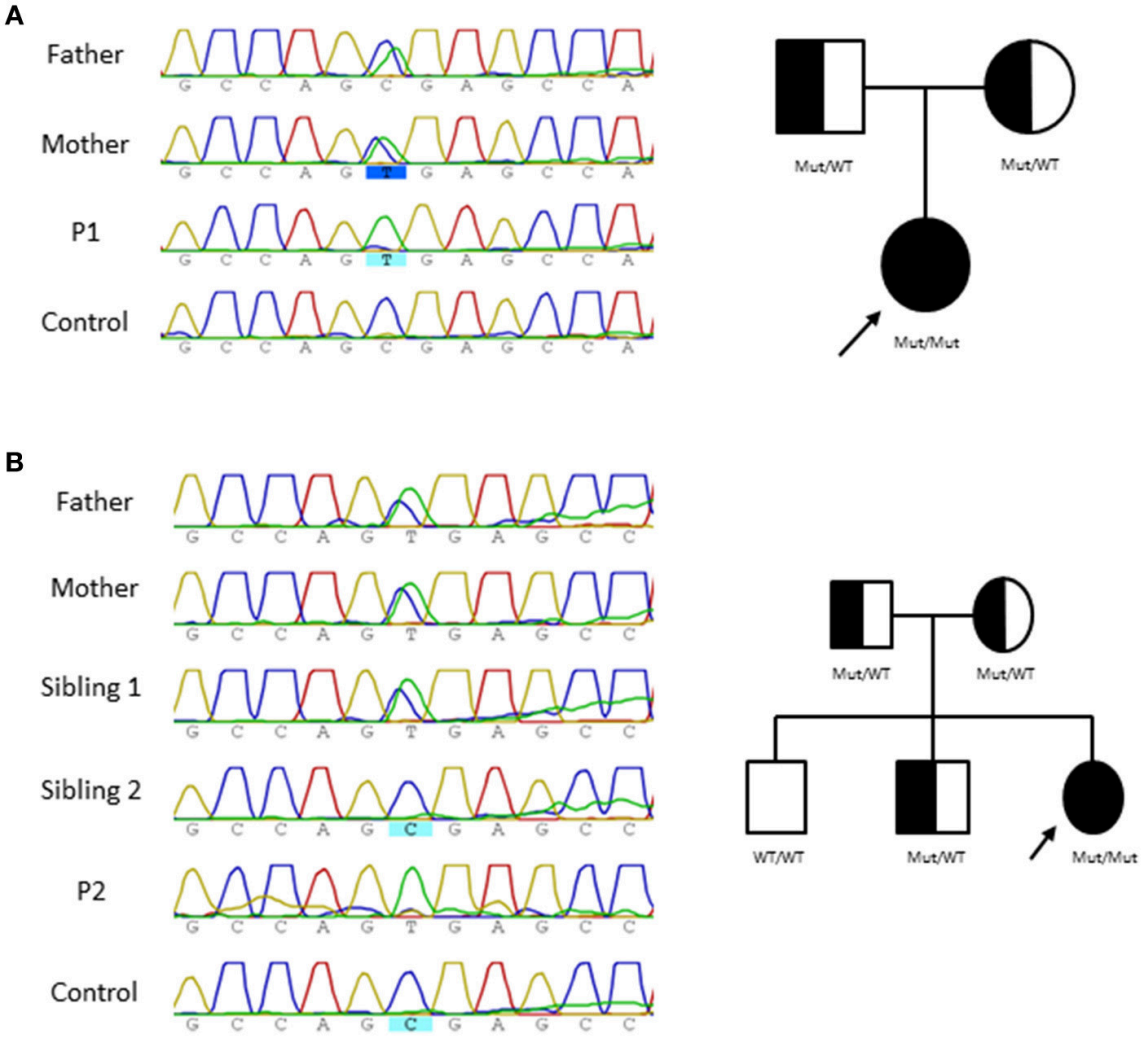
Pedigree and genomic sequence analysis of *NHEJ1* gene. Genomic *NHEJ1* DNA sequence showing c.169C>T mutation and Family tree in P1 **(A)** and P2 **(B)**.

The clinical presentation and immunologic features of both patients lead to suspicion of a primary immunodeficiency (PID). Accordingly, an in house targeted NGS sequencing panel for 192 PID related genes (Table 3) was performed and revealed a homozygous nucleotide substitution in exon 2 of *NHEJ1* gene (NM_024782, c.169C>T) that affects codon 57 and is predicted to result in a severely truncated protein (p.R57X), this mutational change has been previously described in two Cernunnos /XLF defective patients similar to P2 patient displaying microcephaly and slight lymphopenia (1–6). Sanger sequencing confirmed the variants in the patients which were inherited in autosomal recessive fashion from their healthy parents (Figure 1).

**Table 3:** Gene-panel related to PID.

ACT1 ADA AICDA AIRE AK2 AP3B1 AP3D1 ATM BCL10 BLNK BTK C3 CARD11 CARD9 CASP10 CASP8 CD127 CD19 CD20 CD21 CD27 CD3D CD3E CD3G CD3Z CD45 CD79A CD79B CD81 CD8A CEBPE CECR1 CLEC7A COPA CORO1A CTLA4 CTPS1 CTSC CXCR4 CYBA CYBB DCLRE1C DKC1 DNMT3B DOCK2 DOCK8 ELANE EVER1 EVER2 FADD FCGR3A FOXN1 FOXP3 G6PC3 GATA2 GFI1 HAX1 HOIL1 ICOS IFNGR1 IFNGR2 IGHM IGLL1 IKAROS IKBA IKBKB IKBKG IL10 IL10RA IL10RB IL12B IL12RB1 IL12RB2 IL17F IL17RA IL17RC IL1RN IL21 IL21R IL2RA IL2RG IL7 IRAK4 IRF3 IRF7 IRF8 ISG15 ITGB2 ITK JAGN1 JAK3 KIND3 KRAS LAMTOR2 LCK LIG4 LPIN2 LRBA LYST MAGT1 MALT1 MAP3K14 MCM4 MEFV MHC2TA MRE11 MST1 MVK MYD88 NCF1 NCF2 NFKB1 NFKB2 NHEJ1 NHP2 NLRC4 NLRP12 NLRP3 NOD2 NOP10 NRAS ORAI1 p40phox PGM3 PIK3CD PIK3R1 PLCG2 PMS2 PNP POLE1 PRF1 PRKDC PSMB8 PSTPIP1 PTPN6 RAB27A RAG1 RAG2 RFX5 RFXANK RFXAP RLTPR RMRP RNF168 RORC RTEL1 SH2D1A SMARCAL1 SP110 SPINK5 STAT1 STAT2 STAT3 STAT5B STIM1 STX11 STXBP2 TAP1 TAP2 TAPBP TBK1 TCF3 TCN2 TERC TERT TINF2 TIRAP TLR3 TMEM173 TNFRSF13B TNFRSF13C TNFRSF1A TNFRSF5 TNFRSF6 TNFSF5 TNFSF6 TRAF3 TRIF TRNT1 TTC7A TWEAK UNC119 UNC13D UNC93B1 UNG VPS45 WAS WIPF1 XIAP XRCC4 ZAP70 ZBTB24


**Figure 2:**
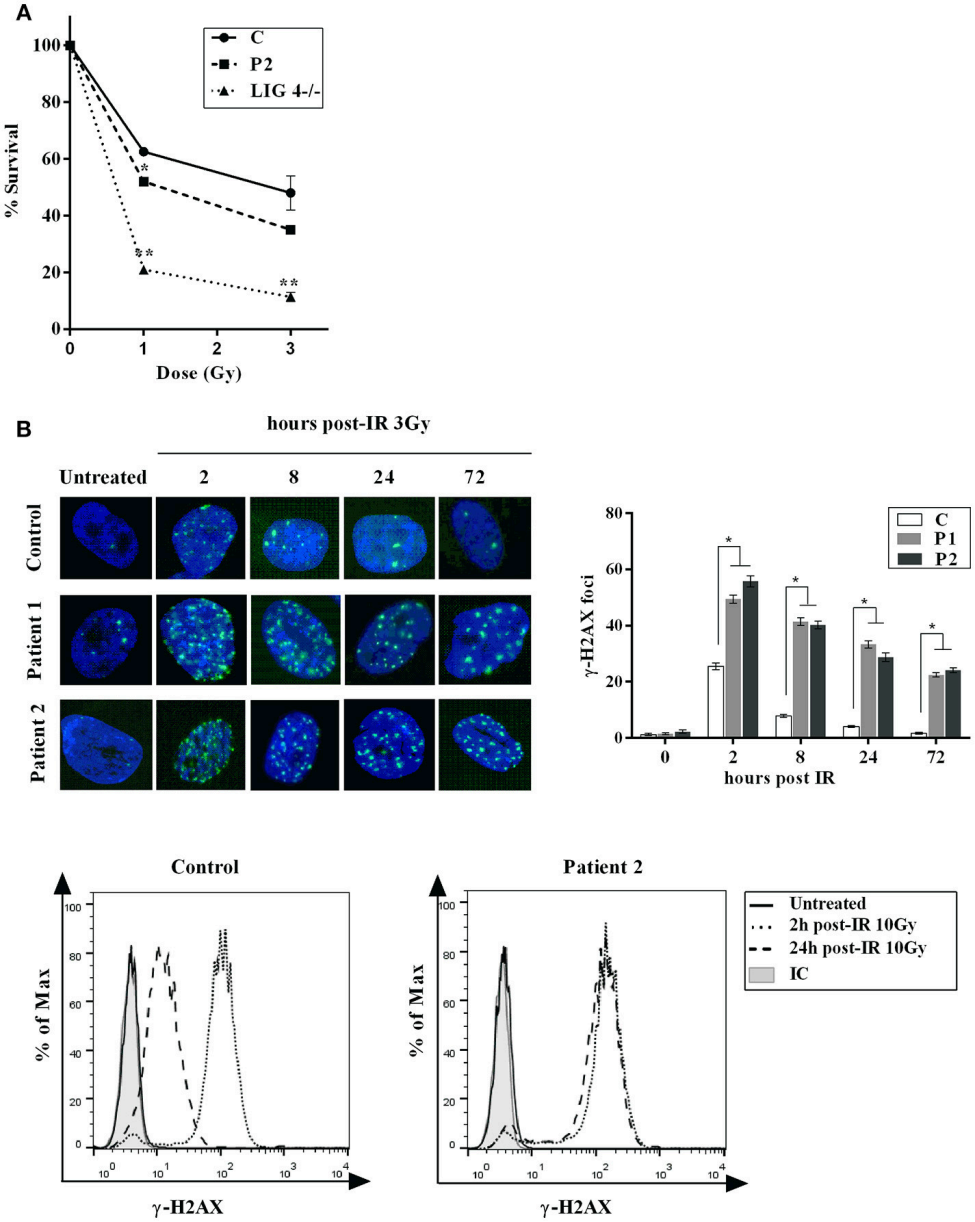
Cellular Response to DNA Damage. **(A)** Clonogenic survival assay in XLF-deficient fibroblasts. Cell survival following IR with γ-rays (1 and 3Gy) was assessed in primary fibroblasts from normal control (C) and patient (P2). LIG4-deficient fibroblasts (LIG4^−/−^) were used as a radiosensitive control. The results represent the mean and standard deviation of two separate experiments and are expressed as percentages of survival cells relative to unirradiated primary fibroblasts. **(B)** Top panel: Primary fibroblasts from control (C) and patients (P1 and P2) were irradiated with 3 Gy and fixed at given time points post-irradiation before staining with anti- γ -H2AX. Nuclei were stained with DAPI. Numbers of γ -H2AX foci per nucleus were determined at indicated time points after irradiation (average number of γ -H2AX foci per nucleus in 30 cells). Error bars represent the SD from 3 independent experiments. Bottom panel: γ-H2AX detection by flow cytometry was performed in PBMCs from P2 and control. Mean fluorescence intensities (MFI) are shown as histograms (unirradiated, 2 and 24 h) compared to isotype (IC). Persistence of γ H2AX signal at 24 h post-treatment in P2 is indicative of a general DNA repair defect. ^*^p < 0.05, ^**^p < 0.01.

Increased sensitivity and decreased double strand breaks (DSB) rejoining to ionizing irradiation (IR) is a feature of Cernunnos /XLF deficient cells. Accordingly, a cell-survival assay revealed an increased IR sensitivity in fibroblasts from P2 similar to that observed in LIG4-deficient fibroblasts (Figure 2A). We also analyze DSB rejoining in primary fibroblasts from both patients (P1 and P2) by enumerating the rate of loss of γ-H2AX foci following exposure to γ-IR. The results showed impaired DSB rejoining after treatment with γ-irradiation and etoposide in both patients (Figure 2B). These findings are in agreement with previously published results in Cernunnos and LIG4-defective patients (7).

Comparative testing of DNA repair was also performed on T cells from the second patient (P2). Upon exposure of PBMCs to 10 Gy, induction and resolution of DNA damage was measured at various time points and a delayed kinetics of DNA repair were observed in P2 compared to a healthy control (Figure 2B).

## Methods

### Cell Culture

Primary fibroblasts were grown in minimal essential medium (MEM) supplemented with 10% fetal calf serum (FCS), penicillin and streptomycin.

### Immunofluorescence and Antibodies

To characterize the repair capacity of the patient's cells we scored the *in situ* modification of the histone variant H2AX, which is phosphorylated proximal to sites of DNA double-strand breaks. The number of phosphorylated H2AX (γH2AX) foci in a nucleus is reported to be directly proportional to the number of DSBs, and de-phosphorylation coincides with DSB repair. Primary skin fibroblasts were irradiated with ionizing irradiation (^137^Cs) or treated with 20 mM Etoposide for 1 h. After the indicated treatments, the slides were washed with PBS, fixed using 4% formaldehyde for 10 min at room temperature and permeabilized with 0.5% Triton X-100 for 5 min. Cells were incubated with primary antibodies for 1 h, the slides were washed with PBS and the bound antibodies were revealed by IgG Alexa fluor antibodies (Invitrogen). Nuclei were counterstained with DAPI, and slides were mounted for immunofluorescence. Images were taken with a fluorescent microscopy (Zeiss AxioImages.A1, Carl Zeiss). In a single experiment at least 30 cells per sample were counted.

### Survival Assay

Primary skin fibroblasts were irradiated with ionizing radiation (^137^Cs). After irradiation, the cells were seeded at a density of 1 × 10^4^ cells/mL in T75 flasks in triplicate. To evaluate cell sensitivity to γ-IR (1 and 3 Gy), adherent cells were trypsinized and counted 11 days later.

### Flow Cytometry

Proportions and lymphocyte count of T-, B-, and NK-cells were determined in blood samples using conjugated mouse anti-human monoclonal antibodies and data were collected by flow cytometry using a Navios Cytometer (Beckman Coulter, Madrid, Spain) and analyzed with Kaluza 1.5a software (Beckman Coulter, Indianapolis IN, US).

PBMCs from patient and healthy controls were irradiated with 10Gy, fixed and stained for CD3, CD19, and phospho-histone H2AX. Mean fluorescence intensities (MFI) of γH2AX were evaluated on gated CD3+ lymphocytes.

### Immunoglobulins

Total serum immunoglobulins (IgG, IgA, IgM, and IgE) were measured by nephelometry (Beckman Coulter, Madrid, Spain).

### NGS and Sanger Sequencing

Genomic DNA was extracted from EDTA whole blood using a MagNa Pure Compact Nucleic Acid Isolation Kit (Roche, Madrid, Spain). Missense *NHEJ1* mutation was identified by targeted next-generation sequencing with an in-house designed panel of 192 genes involved in primary immunodeficiencies (PID) (Ampliseq, Thermo Fisher, Madrid, Spain) and confirmed by PCR and Sanger sequencing using an ABI PRISM 3130 genetic analyzer.

### TRECs and KRECs

TRECs, KRECs, and beta-actin (ACTB) copy numbers were determined from dried blood spots (DBS, punches of 3.2 mm) using triplex real-time quantitative polymerase chain reaction (RT-PCR) (TIB MOLBIOL) and run in a Light Cycler 480 II from Roche Diagnostics.

### Ethics Statement

The protocols of this study were approved by the Institutional Review Board of Hospital Universitario 12 de Octubre (Madrid, Spain) and written informed consent was obtained from all subjects/caregivers in accordance with the Declaration of Helsinki.

## Background

DNA non-homologous end-joining (NHEJ) is the major DNA double strand break (DSB) repair pathway in mammalian cells. NHEJ also functions during immune development rejoining the programmed DSBs introduced during V(D)J recombination (7). Most of the patients deficient in NHEJ components display radiosensitivity and severe combined immunodeficiency (SCID), a phenotype which has been called radiosensitive SCID (RS-SCID). To date, mutations in five genes encoding components of the NHEJ pathway, *LIG4* (encoding DNA ligase IV), *NHEJ1* (encoding Cernunnos), *PRKDC* (encoding DNA-PKcs), *DCLRE1C* (encoding Artemis), and *XRCC4* (encoding XRCC4) have been identified in patients. Although RS-SCID represents the extreme phenotype, radiosensitivity coupled with variable immunodeficiency ranging from Omenn's Syndrome to CID has been observed. Additional features are also observed in some patients, including microcephaly and severe growth delay. However, all of these patients are at risk of malignancies, particularly lymphoma (6).

### History

Cernunnos, was identified through both cDNA complementation of cells derived from a IR-sensitive immunodeficient patient (1) and through a yeast two-hybrid screen for XRCC4-interacting partners (8). Cernunnos is a homolog of Nej1, one of the NHEJ factors identified in yeast (9, 10). Data from several studies suggest that although Cernunnos may not be strictly required for NHEJ, the loss of Cernunnos does affect NHEJ and interestingly DNA repair defect in these patients is quite similar to that found in LIG4 syndrome patients (11).

### Review of Similar Cases

Cernunnos/XLF deficiency in human results in extreme sensitivity to IR, microcephaly, and growth retardation, but the effect on the immune system is variable (1–4, 8). Mutations in *NHEJ1* have been described previously in 27 patients with clinical features comparable to LIG4 deficiencies. Of contrast, XRCC4 deficiency shares the radiosensitivity and neurological impairment but not overt immunodeficiency (5, 12, 13).

Most of the Cernunnos/XLF deficient patients are hypersensitive to IR and have a significant NHEJ defect (1, 14) similar to LIG4 deficiency, which belong to NHEJ ligation complex; immunodeficiency, however, is milder compared with defects in NHEJ factors involved in hairpin opening as Artemis and DNA-PKcs.

In this study, we reported two patients who present the same c.169C>T mutation in *NHEJ1* gene but different immunologic features (Table 2). P2 presented with mild T lymphopenia, hypersensitivity and NHEJ repair defect, typical for patients with Cernunnos/XLF defects (1, 5). On the other hand, P1 presented a more severe phenotype (T-B-); hypersensitivity and NHEJ repair defect, however, was similar to P2. These findings indicate that patients with same *NHEJ1* mutation and DNA repair defect may show great variability in the clinical phenotype.

Patients with homozygous mutations (p.R178X) in *NHEJ1* gene have been previously reported. Two patients died at 1.5 and 4 years (1, 2), while another of the patients remains alive at the age of 8 years (without HSCT) (5). However, none of these patients presented the severe T lymphopenia observed in our first patient.

One explanation for the different degree of lymphopenia found in Cernunnos-deficient patients (P1 and P2) might be the existence of alternative DNA repair proteins that only work to repair DSBs generated during lymphocyte development (3). It has been described that RAG complex and Cernunnos functionally overlap in the repair of DNA breaks during antigen receptor assembly ensuring stabilization of DNA ends after DNA cleavage by RAG (15). ATM and/or ATM-dependent DRR factors, as 53BP1, would contribute to the RAG-DSB stabilization and the recruitment of NHEJ pathway proteins. Severely impaired joining of RAG-generated DSBs in cells that are deficient for Cernunnos and either ATM, 53PBP1or H2AX has been observed (16). Thus, genetic factors such as mutations or polymorphisms in some of these proteins could affect the VDJ recombination process and explain the severe T lymphopenia observed in P1. As Cernunnos is not usually found in the context of a patient with a SCID phenotype we cannot rule out a digenic cause (particularly 53BP1, as other VDJ recombination genes were wild-type in our panel).

## Discussion

### Diagnosis and Treatment

The assignment of a timely and accurate diagnosis is of paramount importance in the management of patients with defects in DNA repair, as HSCT is the only curative therapy available. Usually the repair defect in these disorders is assessed by immunofluorescence assays of irradiation-induced γ-H2AX foci using skin fibroblasts. Flow cytometry (FC) can be applied as a rapid diagnostic tool for DNA repair disorders (17, 18). Therefore, we have used flow cytometry to analyze PBMCs from P2 and the results showed a DNA repair defect similar to that obtained in skin fibroblasts (data not shown) (19).

Hence, a high throughput, sensitive and reliable assay to quantify γ-H2AX foci in PBMCs isolated from blood samples would be a valuable tool to diagnose these patients and thus allow HSCT without delay.

In addition, it would also be helpful in cancer patients to individualize and to guide the dosing of ionizing radiation (IR) and/ or genotoxic agents to avoid accumulation of cells with genomic instability that could accelerate cancer development.

In the era of newborn screening an abnormal TREC assay should be followed by NGS approach as Cernunnos/XLF deficiency may present early in life as SCID, as other RS-SCID defects (20). Since genetic diagnosis takes time, functional radiosensitivity assays in peripheral blood may lead to the correct diagnosis and avoid exposure to alkylating agents during the conditioning regimen even in the absence of a genetic diagnosis (21).

The patients presented in this work are alive and well and both patients after undergoing HSCT. Of note, P2 has survived to age 7 years. It has been reported that the Cernunnos/XLF deficient patients may survive the first years of life, or even up to 18 years, without HSCT. However, due to the comorbilities that these patients face, it is highly recommended HSCT pre-emptively, rather than expect the appearance of a malignant refractory disease disregarding the age at diagnosis (21).

Given the extreme rarity of the disorder, the appearance of two unrelated homozygous cases in non-consanguineous family with the same mutation seems unlikely. However, we can offer three reasons to discard consanguinity in both kindreds: First. Geographical: Both families are more than 450 km apart each other. Both kindreds deny any consanguinity (even far) ties. Second, Molecular: HLA typing were done in both families. It is shown HLA haplotypes generated by segregation analysis in both families: Family 1, Father: A^*^30, B^*^18, DRB1^*^03 and A^*^02, B^*^40, DRB1^*^08; Mother: A^*^01, B^*^37, DRB1^*^13 and A^*^-, B^*^57, DRB1^*^07. Family 2, Father: A^*^02, B^*^39, DRB1^*^11 and A^*^29, B^*^51, DRB1^*^04; Mother: A^*^11, B^*^40, DRB1^*^04 and A^*^11, B^*^07, DRB1^*^15. In conclusion, both families did not share any HLA haplotype (Table 4). Third, No other mutations in PID genes that associate radiosensibility (*LIG4, PRKDC, DCLRE1C, ATM, RNF168*) were found.

**Table 4:** HLA typing in P1 and P2 families.

**HLA**	**P1 Family**			**P2 Family**		
	**P1**	**Father**	**Mother**	**P2**	**Father**	**Mother**
HLA-A	A*01, A*30	A*02, A*30	A*01, A*Null	A*02, A*11	A*02, A*29	A*11, A*11
HLA-B	B*18, B*37	B*18, B*40	B*37, B*57	B*07, B*39	B*39, B*51	B*07, B*40
HLA-DRB1	DRB1*03, DRB1*13	DRB1*03, DRB1*08	DRB1*07, DRB1*13	DRB1*11, DRB1*15	DRB1*04, DRB1*11	DRB1*04, DRB1*15

## Concluding Remarks

We report two unrelated cases diagnosed with Cernunnos/XLF deficiency. Both patients showed a similar DNA repair defect and increased cellular sensitivity to ionizing radiation as per *in vitro* assays. However, clinical presentation and immunological profile were extremely different. The first patient showed a senescent phenotype with decreased TRECs, RTE and naive T-cells counts suggesting that the sustained self-renewal of T-cell pool was impaired, while P2 presented only a slight reduction in T-cell counts as it has been reported in Cernunnos/XLF deficient patients reported up to date with normal TRECs/KRECs and IgG levels (22).

## Author Contributions

JB-E and RR-G performed the laboratory work for this study and computational predictions. MR, RR-G, ML-N, RC-G, MC-P, MP, and JB-E performed the laboratory work for this study. CR, CM, ND-P, NS-R, CR, and LG-G were responsible for the clinical management of the patients. LA, MR, JB-E, and LG-G designed the research and drafted the manuscript. All authors approved the final version of this manuscript.

### Conflict of Interest Statement

The authors declare that the research was conducted in the absence of any commercial or financial relationships that could be construed as a potential conflict of interest.
